# Too Old for Computers? The Longitudinal Relationship Between Stereotype Threat and Computer Use by Older Adults

**DOI:** 10.3389/fpsyg.2020.568972

**Published:** 2020-10-02

**Authors:** João Mariano, Sibila Marques, Miguel R. Ramos, Filomena Gerardo, Hein de Vries

**Affiliations:** ^1^Instituto Universitário de Lisboa (ISCTE-IUL), CIS-IUL, Lisbon, Portugal; ^2^Care and Public Health Research Institute (CAPHRI), Maastricht University, Maastricht, Netherlands; ^3^University of Birmingham, Birmingham, United Kingdom; ^4^Santa Casa da Misericórdia de Lisboa, Lisbon, Portugal; ^5^Instituto Universitário de Lisboa (ISCTE-IUL), DINÂMIA’CET-IUL, Lisbon, Portugal

**Keywords:** aging, ageism, stereotype threat, computer use, longitudinal study

## Abstract

Besides having lower rates of technology adoption than the general population, older adults are commonly stereotyped as lacking technological ability. Stereotype threat, the fear of confirming negative stereotypes targeting their social group, may lead individuals to distance themselves from the stereotyped domain. This suggests that older adults may underuse computer technology due to stereotype threat. A sample of 86 community-dwelling older adults (*M*_age_ = 78.47, SD_age_ = 7.92) participated in a two-wave longitudinal study aiming to examine the relationship between stereotype threat and computer use in this age group. An autoregressive cross-lagged panel analysis was conducted using structural equation modeling. As expected, stereotype threat predicted lower levels of computer use a year and a half later. In turn, computer use was unrelated to the later experience of stereotype threat in this domain. These findings suggest that stereotype threat may undermine computer adoption in late adulthood, thus contributing to perpetuate the digital inequalities between younger and older generations.

## Introduction

Older adults continue to lag behind the general population when it comes to using information and communication technology (König et al., 2018; Organisation for Economic Co-operation and Development, 2019). Unsurprisingly, they are also stereotyped as having less technological ability than younger age groups. Some studies suggest this is particularly common in relation to computer technology. Computer-related activities, such as buying a personal computer and taking a computer course, are seen as requiring high competence but also as being less typical of older individuals (Ryan and Heaven, 1988; Ryan et al., 1992). Compared to younger peers, older adults are perceived as less likely to take and to complete a computer course, and failing the course is more attributed to their age (Ryan et al., 1992). In the workplace, older employees are considered less experienced and less comfortable with computer technology (Hanks and Icenogle, 2001; McGregor and Gray, 2002).

Although they may partly stem from actual age differences in computer performance and usage, these stereotypes may act as self-fulfilling prophecies (Allport, 1954). Numerous studies have repeatedly shown that stereotyped group members tend to behave in stereotype consistent ways (for reviews, see Wheeler and Petty, 2001; Meisner, 2011), suggesting that ageist stereotypes about computer competence may lead older adults to underuse computer technology. This stresses the importance of exploring age stereotypes as possible barriers to computer use in this age group, as this overlooked factor may keep older individuals from taking advantage of its potential benefits to their health and well-being (Heo et al., 2015; Hartanto et al., 2020).

The present study aimed to investigate the longitudinal relationship between stereotype threat and computer use in late adulthood. Stereotype threat is the concern or worry about confirming, or being seen to confirm, negative stereotypes about the group to which one belongs (Steele, 1997; Steele et al., 2002). Research has focused primarily on its immediate impact on task performance. Stereotype threat can cause stereotyped group members to unintentionally underperform in stereotype relevant tasks. This has been documented across different social groups and ability domains, including minorities in academics (e.g., Steele and Aronson, 1995) and women in mathematics (e.g., Spencer et al., 1999). Among older adults, stereotype threat has been found to impair performance across multiple cognitive and physical tasks (for a review, see Lamont et al., 2015).

Despite receiving considerably less theoretical and empirical attention, another behavioral response to stereotype threat is to avoid or abandon the domain where the stereotype applies (Steele et al., 2002). Individuals may distance themselves from situations or activities in which they risk confirming negative stereotypes targeting their group. Avoidance can be an acute, short-term reaction to stereotype threat. For example, women exposed to gender-stereotypic television commercials subsequently avoided math items in favor of verbal items on an aptitude test (Davies et al., 2002) and avoided assuming leadership roles in favor of subordinate roles (Davies et al., 2005). Likewise, avoidance and abandonment may result from chronic, long-term exposure to stereotype threat. For instance, stereotype threat experienced by racial/ethnic minority students predicted lower intention to pursue and actual engagement in a scientific career years later (Woodcock et al., 2012, 2016). In the workplace, stereotype threat has been associated with higher intentions to resign and retire among older employees (von Hippel et al., 2013, 2019).

Applied to the technological domain, one would expect stereotype threat to cause older adults to underuse computers. With the growing digitalization of everyday life (Organisation for Economic Co-operation and Development, 2019), older individuals are increasingly expected and required to interact with computer technology to accomplish their daily activities and responsibilities, for example, when accessing health and public services (eHealth and eGovernment), and may often experience the threat of confirming negative stereotypes about the technological ability of their age group. A likely defense strategy may be to avoid interacting with computers. Over time, the repeated experience of stereotype threat may compromise the adoption or regular use of computer technology. The possibility that older adults may simply avoid engaging with computers due to stereotype threat emphasizes the relevance of looking beyond its short-term impacts on task performance and understand its long-term implications.

Although some studies have examined stereotype threat in the computer domain, none have explored its lasting impact on use behavior. Koch et al. (2008) found that female students attributed their failure in a computer-related task to their own inability after being exposed to the stereotype that men outperform women in computer tasks. Although this study did not examine behavioral outcomes, these internal attributions may negatively influence computer self-efficacy and in turn computer adoption. Furthermore, Fritzsche et al. (2009) explored stereotype threat effects on older adults’ training performance on a computerized library task. Contrary to their predictions, those in the stereotype threat condition performed better than those in the control condition, possibly because the training intervention between the threat manipulation and performance measurement may have motivated participants to disconfirm the stereotype. Besides examining short-term performance rather than long-term use, this study focused on age stereotypes about learning ability, another prevalent stereotype about older adults (Posthuma and Campion, 2009), making it difficult to disentangle from stereotype threat effects specifically associated with the computer inability stereotype. Overall, these findings are insufficient to conclude whether the detrimental effects on behavior commonly associated with the experience of stereotype threat also apply to older individuals in the computer domain.

The present study sought to understand whether concerns about confirming negative stereotypes may compromise older adults’ behavioral engagement with computer technology by examining the longitudinal relationship between stereotype threat and computer use in this age group. Most studies exploring avoidance and abandonment as long-term consequences of stereotype threat have relied on behavioral intention as an indicator of actual behavior or employed cross-sectional designs that prevent inferences about directionality and causality (von Hippel et al., 2011, 2013; Woodcock et al., 2012; Smith et al., 2015). A longitudinal design was chosen to address these limitations and elucidate the temporal and directional relationship between stereotype threat and computer use. Additionally, we focused on desktop and laptop computers, which tend to be perceived as more difficult to use than other types of computerized technologies, such as tablets (Tsai et al., 2015). Because stereotype threat effects on behavioral performance are more pronounced on difficult tasks (Barber et al., 2020), they may be particularly detrimental with regards to desktop and laptop computer use behavior.

A community sample of individuals aged 60 years or older completed measures of stereotype threat and computer use at two time points a year and a half apart. We hypothesized that stereotype threat in the computer domain (Time 1 [T1]) would significantly predict lower levels of computer use a year and a half later (Time 2 [T2]). Additionally, we examined the relationship between computer use at T1 and stereotype threat at T2, although no specific hypothesis was proposed. To the best of our knowledge, this is the first longitudinal study to investigate the long-term consequences of stereotype threat among older adults (for reviews, see Lamont et al., 2015; Barber, 2020).

## Materials and Methods

### Participants

Eligibility criteria included being 60 years or older and living independently in the community. A convenience sample of 114 community-dwelling older adults was recruited across six senior centers in Lisbon, Portugal. Our analysis focused on 86 participants (62 females and 24 males) who completed both phases of the study (retention rate of 75.44%). Their age ranged from 62 to 95 years (*M* = 78.47, SD = 7.92) and their education ranged from 0 to 19 years (*M* = 5.10, SD = 3.12). Most participants lived alone (53.49%, *n* = 46) or with their spouse (25.58%, *n* = 22). The vast majority of participants were retired (97.67%, *n* = 84).

### Procedure

This study complied with institutional and international ethical standards for research involving human participants (Center for Social Research and Intervention, 2013; American Psychological Association, 2017). A local charity in the city of Lisbon and six of its senior centers collaborated in the study and approved the research protocol. Individuals attending the senior centers were invited to participate in a study about computer technology. After providing their informed consent, 114 participants completed a baseline questionnaire (T1). A year and a half later, 86 of those participants completed a follow-up questionnaire (T2). In each senior center, data collection took place in a quiet room, individually, and with the assistance of a researcher whenever necessary. At both time points, participants completed paper-and-pencil questionnaires containing measures of stereotype threat, computer use, and demographics, as well as other measures that were not subject to analysis in the present study.

### Measures

#### Stereotype Threat

Three items from Marx and Goff (2005) and Steele and Aronson (1995) were adapted to assess stereotype threat in the computer domain: “I worry that my ability to perform well using computers is affected by my age,” “I worry that I am unable to use computers because of my age,” “I worry that people feel I am less able to use computers because of my age.” Participants responded on a five-point scale: 1 = *strongly disagree*, 2 = *disagree*, 3 = *neither agree nor disagree*, 4 = *agree*, 5 = *strongly agree*. A higher score indicated a greater experience of stereotype threat (Cronbach’s alpha: α_T__1_ = 0.82, α_T__2_ = 0.87). Measures at T1 and T2 were strongly correlated, suggesting good retest reliability (*r* = 0.51, *p* < 0.001). Item scores at each time point were averaged for the preliminary and descriptive analyses.

#### Computer Use

Desktop or laptop computer use was assessed on two dimensions: frequency and duration (Venkatesh et al., 2008). Frequency of use (“How frequently do you use desktop or laptop computers?”) was rated on a six-point scale (Davis, 1989): 1 = *never*, 2 = *less than once a week*, 3 = *once a week*, 4 = *several times a week*, 5 = *once a day*, 6 = *several times a day*. Duration of use (“How many hours a week do you use desktop or laptop computers?”) was rated on a six-point scale (Czaja et al., 2006): 1 = *never*, 2 = *less than 1 hour a week*, 3 = *between 1 hour and 5 hours a week*, 4 = *between 6 hours and 10 hours a week*, 5 = *between 11 hours and 15 hours a week*, 6 = *more than 15 hours a week*. A higher score indicated a greater level of computer use (Spearman-Brown coefficient: ρ_T__1_ = 0.96, ρ_T__2_ = 0.97). This measure had good retest reliability (*r* = 0.56, *p* < 0.001). Item scores were averaged for the preliminary and descriptive analyses.

#### Covariates

Participants also reported their age, education, sex, living arrangements, occupational status, health status, and computer experience. Health status (“How do you rate your health in general?”) was rated on a seven-point scale ranging from 1 (*terrible*) to 7 (*excellent*). Prior experience with desktop or laptop computers (“How long have you been using desktop or laptop computers?”) was rated on a six-point scale (Czaja et al., 2006): 1 = *never*, 2 = *less than 6 months*, 3 = *more than 6 months, but less than 1 year*, 4 = *more than 1 year, but less than 3 years*, 5 = *more than 3 years, but less than 5 years*, 6 = *more than 5 years*.

## Results

### Preliminary Analysis

A logistic regression was performed to examine potential differences between those who completed the study and those who dropped out. The dependent variable was coded 1 (*baseline and follow-up*) and 0 (*baseline only*). All covariates, stereotype threat, and computer use assessed at baseline were included as independent variables. None of these variables significantly predicted participation in both waves.

### Descriptive Analysis

Table 1 presents the means, standard deviations, and correlations for all measures. Stereotype threat scores approached the midpoint of the scale (*M*_T__1_ = 2.90, *M*_T__2_ = 2.72), suggesting that participants experience moderate levels of stereotype threat in the computer domain. In turn, computer use scores were relatively low (*M*_T__1_ = 1.72, *M*_T__2_ = 1.46). Differences in the study variables across the two time points were examined with repeated measures analysis of variance (ANOVA). Only computer use differed significantly between waves, *F* (1, 85) = 4.91, *p* = 0.029, indicating lower levels of computer use at T2 compared to T1.

**TABLE 1:** Means, standard deviations, and correlations between variables at both time points.

Variable	*M*	SD	1	2	3
1. Stereotype threat (T1)	2.90	1.30	–		
2. Stereotype threat (T2)	2.72	1.35	0.51***	–	
3. Computer use (T1)	1.72	1.23	−0.32**	–0.16	–
4. Computer use (T2)	1.46	1.05	−0.36***	−0.32**	0.56***

T1 = Time 1, T2 = Time 2. **p < 0.01; ***p < 0.001.

### Longitudinal Analysis

Following the analytical approach recommended by Little et al. (2007), an autoregressive cross-lagged panel design was used to examine the relationship between stereotype threat and computer use across two time points. Structural equation modeling (SEM) was performed with Mplus 8 (Muthén and Muthén, 1998-2017) using robust maximum likelihood estimation (MLR), which provides standard errors and chi-square statistics that are robust to non-normality. Model fit was examined based on the Chi-Square Test (χ^2^), the Comparative Fit Index (CFI), the Tucker-Lewis Index (TLI), the Root Mean Square Error of Approximation (RMSEA), and the Standardized Root Mean Square Residual (SRMR). CFI and TLI values of 0.90 or above and RMSEA and SRMR values of 0.08 or below were considered indicative of acceptable fit (Browne and Cudeck, 1993; Hu and Bentler, 1999).

As the first step of the longitudinal analysis, we tested the measurement model. Stereotype threat and computer use at two time points were modeled as latent factors with their respective items serving as observed indicators. The residuals of corresponding indicators were correlated across waves. To ensure that the same constructs were measured across time, longitudinal factorial invariance was tested by comparing an unconstrained model with a constrained model in which the factor loadings of corresponding indicators were equated across waves (Little et al., 2007). A Satorra-Bentler scaled chi-square difference test revealed no significant difference between the models, Δχ^2^ (3) = 1.51, *p* = 0.681, demonstrating weak factorial invariance. A comparison of all fit indices with their recommended values suggested a good fit between the measurement model and the data: χ^2^ (27) = 25.88, *p* = 0.525, CFI = 1.00, TLI = 1.00, RMSEA = 0.00 (90% Confidence Interval (CI) [0.00, 0.08]), SRMR = 0.05.

**FIGURE 1:**
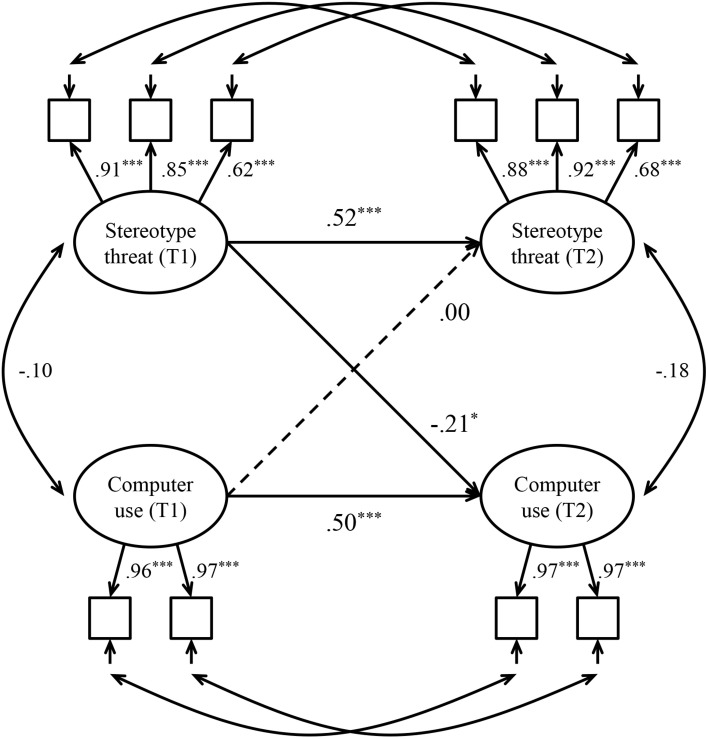
Autoregressive cross-lagged panel model testing the relationship between stereotype threat and computer use. All path coefficients are standardized. Dotted lines indicate non-significant paths. Age, education, sex, living arrangements, occupational status, health status, and computer experience were included as covariates (omitted for clarity). T1 = Time 1, T2 = Time 2. ^∗^*p* < 0.05; ^∗∗∗^*p* < 0.001.

As the second step, we tested the structural model. Autoregressive paths were specified between each variable at T1 and the same variable at T2. Cross-lagged paths were defined between each variable at T1 and the other variable at T2. Age (in years), education (in years), sex (1 = *female*), living arrangements (1 = *alone*), occupational status (1 = *retired*), health status, and computer experience reported at baseline were included as covariates, as they are known predictors of computer use by older adults (Wagner et al., 2010). The structural model revealed a satisfactory fit to the data: χ^2^ (83) = 112.87, *p* = 0.016, CFI = 0.95, TLI = 0.94, RMSEA = 0.07 (90% CI [0.03, 0.09]), SRMR = 0.06. Figure 1 presents the autoregressive cross-lagged panel model with standardized path coefficients and significance levels. The autoregressive effects of stereotype threat (β = 0.52, *p* < 0.001) and computer use (β = 0.50, *p* < 0.001) were significant, suggesting the stability of these constructs. Supporting our hypothesis, the cross-lagged effect of stereotype threat (T1) on computer use (T2) was significant and negative (β = -0.21, *p* = 0.017), implying that higher levels of stereotype threat precede lower rates of computer use. In turn, the cross-lagged effect of computer use (T1) on stereotype threat (T2) was non-significant (β = 0.00, *p* = 0.982), suggesting that computer use is unrelated to the later experience of stereotype threat.

## Discussion

Based on the assumption that stereotype threat leads negatively stereotyped group members to avoid or abandon domains where the stereotype applies (Steele et al., 2002), the present study examined whether older adults underuse computer technology due to stereotype threat. As expected, stereotype threat subsequently predicted lower levels of computer use a year and a half later. This suggests that older adults avoid using computer technology due to the threat of confirming the negative stereotype that their age group lacks computer ability. By contributing to the lower rates of technology adoption in this population, stereotype threat may deprive older adults from its potential benefits and exclude them from this growing dimension of everyday life. This supports the notion that stereotype threat has far reaching detrimental consequences in late adulthood (Barber, 2020), as digitally excluded older adults may face greater difficulties, for example, in accessing health information and services, establishing and maintaining social relationships, and accessing employment and training opportunities (Cotten, 2017).

Another important and novel contribution to stereotype threat research is the finding that computer use did not predict stereotype threat a year and a half later. This suggests that individuals may experience stereotype threat regardless of their prior behavioral engagement with the stereotyped domain. In fact, avoiding or abandoning the domain may not prevent one from continuing to experience stereotype threat. This is consistent with the argument that anyone can potentially experience stereotype threat, as long as one belongs to a negatively stereotyped group (Steele et al., 2002). Whether they are heavy users, light users, or non-users of computer technology, older adults may still worry about confirming ageist stereotypes about the computer ability of their age group. Importantly, this finding suggests that interventions aiming to promote computer use will not attenuate the experience of stereotype threat in this domain.

This study addressed several limitations of past research exploring the long-term consequences of stereotype threat on behavioral outcomes. Firstly, previous studies focused mainly on intention rather than behavior (von Hippel et al., 2011, 2013; Woodcock et al., 2012; Smith et al., 2015). The few studies exploring behavior used dichotomous measures of complete engagement or abandonment of the threatening domain (Beasley and Fischer, 2012; Woodcock et al., 2016). In contrast, we relied on a bidimensional measure that conceptualized computer use in terms of frequency and duration (Venkatesh et al., 2008). Secondly, although prior studies have reliably shown a negative relationship between stereotype threat and behavioral intention, many of them used cross-sectional designs (von Hippel et al., 2011, 2013; Smith et al., 2015), which precludes the establishment of directionality and causality. By using an autoregressive cross-lagged panel design, we were able to describe the directional influence between variables. Our findings suggest that, although stereotype threat subsequently predicted computer use, use behavior did not influence the later experience of stereotype threat. Lastly, most of these studies have focused on gender- and race-based stereotype threat either on academic or organizational domains (von Hippel et al., 2011; Woodcock et al., 2012, 2016; Smith et al., 2015). The few studies focusing on age-based stereotype threat explored its impact in the workplace (von Hippel et al., 2013, 2019). Innovatively, we examined the longitudinal consequences of stereotype threat among older adults in the computer domain, an understudied domain in stereotype threat research.

Future studies should explore whether stereotype threat effects on computer task performance may impact computer use behavior in the long run. Similarly to other domains in which they are negatively stereotyped (Lamont et al., 2015), stereotype threat should disrupt the computer performance of older adults. Computer task performance can influence attitudes toward computers, including computer interest (Czaja and Sharit, 1998). This suggests that, by disrupting older adults’ performance, stereotype threat may indirectly compromise their willingness to use computers in the future. Nevertheless, the experience of stereotype threat may undermine computer use and adoption by causing older adults to simply avoid situations in which they risk confirming the stereotype, that is, to avoid any possibility of performance.

The specific tasks or activities that older adults perform when using computers should also be examined in future research. Rather than avoiding computers completely, older adults may resist performing new or unfamiliar tasks for fear of confirming they lack the necessary ability. If older adults avoid using computers due to stereotype threat as our study suggests, this experience should be associated with a limited range of computer activities, in line with evidence that older individuals use computers for fewer activities compared to younger age groups (Czaja et al., 2006). Another possibility is that stereotype threat may lead older adults to distance themselves from computers in favor of other types of technology. For instance, tablets tend to be perceived as easier to use than computers (Tsai et al., 2015), suggesting that older adults may be less likely to worry about confirming age stereotypes about technological inability when they consider using them. This is consistent with the increasing rates of mobile device adoption in the older population (Anderson and Perrin, 2017) and the decreasing levels of computer use over a year and a half in our sample. We were unable to test this possibility in the present study because tablet use levels were very low. Future research should explore these potential processes, while also examining the generalizability of stereotype threat effects to other technological devices.

Vulnerability to stereotype threat in the computer domain should be investigated in specific groups within the older population. Since older workers are stereotypically perceived as less technologically skilled (Van Dalen et al., 2009), they may also experience stereotype threat effects on technology-related behaviors, particularly in the workplace. Likewise, women are negatively stereotyped with regards to their computer competence and there is evidence of gender-based stereotype threat in this domain (Cooper, 2006; Koch et al., 2008). This suggests that older women may be more susceptible to its detrimental effects on computer use, consistently with evidence of stronger age-based stereotype threat effects on task performance in this group (Lamont et al., 2015).

Our findings highlight the importance of developing effective interventions to counter stereotype threat effects on computer use behavior. Experimental evidence has shown that informing individuals about stereotype threat (Mazerolle et al., 2016) and promoting either experienced or imagined intergenerational contact (Abrams et al., 2006, 2008) reduced the negative effects of stereotype threat on older adults’ cognitive performance. The latter approach may be particularly promising, as intergenerational contact can also improve stereotypes and attitudes toward older people (for reviews, see Burnes et al., 2019; Marques et al., 2020). Still, intergenerational programs focusing on technology use must be carefully designed, as they may end up reinforcing stereotypical perceptions of older adults as incompetent (Drury et al., 2017). Future studies should identify the best strategies to effectively prevent the lasting impact of stereotype threat in the technology domain in order to bridge the digital divide between generations.

## Data Availability Statement

The data supporting the conclusions of this study will be made available upon request to the corresponding author.

## Ethics Statement

This study complied with institutional and international ethical standards for research involving human participants. Institutional and legal approval for the study and its research protocol was obtained from the legal office of the charity that collaborated in the study. All participants provided their written informed consent to take part in the study.

## Author Contributions

All authors contributed to this work and approved the submitted version. JM contributed to the research conception, study design, data collection, statistical analysis, and writing of the manuscript. SM contributed to the research conception, study design, and critical revision of the manuscript. MR contributed to the statistical analysis and critical revision of the manuscript. FG contributed to the data collection. HV contributed to the research conception, study design, and critical revision of the manuscript.

## Conflict of Interest

The authors declare that the research was conducted in the absence of any commercial or financial relationships that could be construed as a potential conflict of interest.
